# Role of Cod Liver Oil in Reducing Elevated Lipid Parameters

**DOI:** 10.7759/cureus.15556

**Published:** 2021-06-09

**Authors:** Faryal Fatima, Asadullah Memon, Shumaila Zafar, Zain Amar, Abdul Subhan Talpur, Sara Hashim, Hamza Maqsood, Farukhzad Hafizyar, Besham Kumar

**Affiliations:** 1 Internal Medicine, Liaquat University of Medical and Health Sciences, Jamshoro, PAK; 2 Emergency Department, Kohsar Hospital, Hyderabad, PAK; 3 Internal Medicine, Mayo Hospital, Lahore, PAK; 4 Internal Medicine, Isra University, Hyderabad, PAK; 5 Pathology and Laboratory Medicine, Bolan Medical College, Quetta, PAK; 6 Internal Medicine, Nishtar Medical University, Multan, PAK; 7 Internal Medicine, Ariana Sabet Hospital, Kabul, AFG; 8 Internal Medicine, Jinnah Postgraduate Medical Centre, Karachi, PAK

**Keywords:** lipid, cholesterol, rosuvastatin, cod liver oil, reduction

## Abstract

Introduction

The health benefits of cod oil, which includes omega-3 fatty acids, have been of considerable interest to medicine due to its promising results. Studies have shown successful therapeutic effects of a high dietary intake of omega-3 fatty acids by reducing the synthesis of very-low-density lipoprotein, with subsequent low levels of serum triglycerides.

Methods

This single-blind placebo-controlled two-arm interventional study was conducted in the internal medicine unit of a tertiary care hospital from October 2020 to April 2021. 600 treatment naïve patients with elevated cholesterol levels and/or elevated low-density lipoprotein (LDL) were enrolled in the study and randomized into two groups. The study group received 415 mg cod liver oil daily as a capsule in a bottle, in addition to 10 mg rosuvastatin. On the other hand, the control group received 10 mg rosuvastatin with placebo capsules in an identical bottle. Participants were followed up on day 30.

Results

There was relatively more significant reductions in the total cholesterol (152.22 ± 29.75 mg/dL vs. 171.65 ± 31.21 mg/dL; p-value: <0.0001) and LDL (72.41 ± 27.52 mg/dL vs. 79.15 ± 29.12 mg/dL; p-value: <0.0001) in the intervention group compared to the placebo group after day 30. There was a significant reduction in all lipid values in both groups at day 30 as compared to day 0.

Conclusion

Our study indicates that cod liver oil in addition to rosuvastatin reduces cholesterol more compared to rosuvastatin alone. However, in all cases, lifestyle changes should be the first modification adopted by the patients. Further large-scale trials are needed to examine the role of cod liver oil in reducing lipid values.

## Introduction

Ingestion of seafood and some marine oils can have a beneficial effect on health. Observation from the past years has revealed that the human population, such as those of Eskimos has a lower incidence of coronary heart disease, attributed to their seafood-rich diet [1]. One such food is cod liver oil. Obtained from fatty fish, it is a source of vitamin A, vitamin D, and omega- 3 polyunsaturated fatty acids. It contains essential fatty acids like eicosapentaenoic and (EPA) and docosahexaenoic acid (DHA), which have a marked influence on the hemostatic function and lipid content of the body [2].

The health benefits of cod oil have been of considerable interest to medicine due to its promising results. Some studies have shown a positive association, while others did not [3]. Various experimental and longitudinal studies have shown successful therapeutic effects of a high dietary intake of omega-3 fatty acids by reducing the synthesis of very-low-density lipoproteins (VLDL), with subsequent low levels of serum triglycerides (TG) [3]. A negative association has also been reported between fish consumption and serum total cholesterol (TC) [3]. Due to its alteration with body lipid contents, the possible beneficial influence of polyunsaturated fatty acids found in dietary fish oil can be of value in preventing atherosclerosis, ischemic heart disease, hypertension, and diabetes mellitus (DM) [4,5].

Several theories have been put forth to explain the mechanism of action of these oils, but no clear hypothesis has been made. It has been found to reduce the hepatic synthesis of VLDL with subsequent changes in the plasma lipid composition [6]. Mean plasma TG concentrations were reduced and those of high-density-lipoprotein (HDL) cholesterol were increased by the supplement. These fatty acids actively incorporate themselves into the cell membrane and decrease membrane lipid fluidity [6]. Some studies have also suggested its anti-atherosclerotic and anti-platelet aggregational effects, explaining its importance in preventing coronary heart disease [7]. It has also been suggested that it prolongs primary bleeding time, leads to low platelet aggregation and low platelet thromboxane A2 synthesis. EPA competitively inhibits the production of prostaglandins and leukotrienes derived from arachidonic acid and may result in a reduction of inflammation via these and other mechanisms [7].

There is limited data locally available that have studied the efficacy of cod liver oil in reducing lipid values. Therefore, in this study, we aim to understand the impact of cod liver oil on lipid profile in our population to reduce the morbidity associated with the increased lipid content of the body.

## Materials and methods

This single-blind placebo-controlled two-arm interventional study was conducted in the internal medicine unit of a tertiary care hospital from October 2020 to April 2021. After obtaining informed consent, 600 treatment naïve patients with elevated cholesterol levels and/or elevated low-density lipoprotein (LDL) were enrolled in the study. Participants with severe liver and kidney disease were excluded from the study. Before enrollment of patients, ethical review board approval was taken from the institute.

 Elevated cholesterol was defined as serum cholesterol levels of more than 200 mg/dl and elevated LDL was defined as serum LDL levels of more than 100 mg/dl [8]. Patients were enrolled using consecutive convenient non-probability sampling techniques. Participants were randomized into two groups by 1:1 ratio using an online randomizer, research randomizer (https://www.randomizer.org/) software. The study group received 415 mg cod liver oil daily as capsules in an unidentified bottle, in addition to 10 mg rosuvastatin. On the other hand, the control group received 10 mg rosuvastatin with placebo capsules in an identical bottle.

The patient’s characteristics such as age, gender, history of smoking, hypertension, type 2 DM, and, body mass index (BMI) were noted in a self-structured questionnaire. Patients were scheduled to return for follow-up on day 30. Blood was drawn via phlebotomy and sent to the laboratory for lipid profile. On their follow-up visit, lipid profile was done again.

Participants in the study group and control group lost to follow-up were 26 and 24, respectively. Only participants who completed the study were included in the final analysis. Statistical analysis was done using the Statistical Packages for Social Sciences (SPSS) IBM Corp. Released 2015. IBM SPSS Statistics for Windows, Version 23.0. Armonk, NY: IBM Corp. Continuous variables such as age and lipid values were analyzed via descriptive statistics and were presented as mean and standard deviation (SD), while categorical variables such as gender, smoking status, hypertension, BMI, and DM were presented by percentages and frequencies. We correlated the means within each group (baseline vs. day 30) by applying a dependent t-test and within the two groups (intervention vs. the placebo group, at day 30) by applying an independent t-test. A p-value of less than 0.05 meant that there is a difference between the two groups and the null hypothesis is not correct.

## Results

No significant difference was found in the general characteristics of the patients treated with and without cod liver oil (Table 1).

**Table 1:** Characteristics of the participants treated with and without cod liver oil BMI: body mass index, DM: diabetes mellitus, NS: nonsignificant

Characteristics	Patients treated with cod liver oil (n= 274)	Patients treated without cod liver oil (n= 276)	p-value
Age in year (Mean ±SD)	51 ± 14	52 ± 14	NS
Male (%)	171 (62.4%)	169 (61.2%)	NS
Hypertension (%)	201 (73.3%)	205 (74.2%)	NS
Smoking (%)	81 (29.5%)	84 (30.4%)	NS
Type 2 DM (%)	101 (36.8%)	105 (38.0%)	NS
BMI greater than 25 kg/m^2 ^(%)	71 (25.9%)	74 (26.8%)	NS

At day 30, there was relatively more significant reductions in the TC (152.22 ± 29.75 vs. 171.65 ± 31.21; p-value: <0.0001) and LDL (72.41 ± 27.52 vs. 79.15 ± 29.12; p-value: <0.0001) in the intervention group compared to the placebo group. There was a significant reduction in all lipid values in both groups at day 30 as compared to day 0 (Table 2).

**Table 2:** Lipid profile of patients treated with and without cod liver oil on day 0 and day 30 HDL: high density lipoprotein, LDL: low density lipoprotein, TC: total cholesterol, TG: triglyceride

Lipid profile (mg/dL)	Patients treated with cod liver oil (n= 274)			Patients treated without cod liver oil (n= 276)			p-value
	Day 0	Day 30	p-value	Day 0	Day 30	p-value	
TC	221.41 ± 34.21	152.22 ± 29.75	<0.0001	222.12 ± 35.11	171.65 ± 31.21	<0.0001	<0.0001
HDL	36.72 ± 10.01	41.12 ± 10.51	<0.0001	36.21 ± 9.51	41.02 ± 9.89	<0.0001	0.908
LDL	158.41 ± 41.02	72.41 ± 27.52	<0.0001	160.12 ± 41.52	79.15 ± 29.12	<0.0001	0.005
TG	143.51 ± 35.25	120.69 ± 32.65	<0.0001	141.51 ± 35.71	122.69 ± 31.98	<0.0001	0.46

## Discussion

In our study, after being examined for 30 days, both groups showed a significant reduction in their TC, LDL, and TG levels. An increase in the levels of HDL was also noted. However, upon the intake of cod liver oil, the interventional group showed a significantly higher reduction in cholesterol and lipid compared to the placebo group.

Evidence supports our findings by proving that daily administration of 40 mg rosuvastatin is an effective statin therapy in terms of lowering LDL up to 63% [9]. Some other comparative trials have also provided data suggesting that rosuvastatin is the most beneficial statin for lowering LDL [10,11]. Its role in increasing HDL levels has been demonstrated in a comparative study in which patients with hypercholesterolemia were enrolled and a dosage of 10-40 mg of rosuvastatin was able to show increased HDL levels by 7.7-9.6% [11]. Rosuvastatin is generally considered a safe drug, but sometimes adverse effects are also observed. Patients using this drug might undergo memory problems. Rare side effects include muscle-related problems, which uncommonly could lead to rhabdomyolysis and autoimmune myopathy. In very few patients, liver and kidney-related problems could develop [12]. Fish oil, with components of omega-3 polyunsaturated fatty acids, has shown efficacy in lowering TG levels, which would in turn reduce the risks of cardiovascular diseases [13]. This has also been demonstrated in our study. Such effects of fish oil are obtained by inhibiting the metabolism of lipid synthesis and increasing the oxidation of fatty acid, particularly in the liver [14-16]. A recent trial showed that EPA (312 mg) formulation reduces cardiovascular events by 25% in statin-treated patients with elevated lipid values [17].

To the best of our knowledge, this is the first study in the local population that has studied the effect of cod liver oil on lipid values. However, since it was a single institute study, care should be taken while inferring the result to a greater population. The results of this study suggest both, cod liver oil and rosuvastatin are beneficial in lowering LDL and TC levels. This could potentially avoid all the diseases related to increased cholesterol and lipid levels, like cardiovascular diseases. Adding cod liver oil to rosuvastatin as a supplement may help reduce the dose of rosuvastatin, which might help in subsiding their adverse reactions. Cod liver oil can also be suggested as an alternative in patients in whom statins are contraindicated, however, clinical evidence is required.

## Conclusions

The results of this study suggest both, cod liver oil and rosuvastatin are beneficial in lowering LDL and TC levels and hence have a beneficial effect in patients with hyperlipidemia. However, in all cases, lifestyle changes should be the first modification adopted by the patients. Further large-scale trials are needed to understand if adding cod liver can allow physicians to lower the dose of rosuvastatin. 

## References

[1] von Schacky C, Fischer S, Weber PC (1985). Long-term effects of dietary marine omega-3 fatty acids upon plasma and cellular lipids, platelet function, and eicosanoid formation in humans. J Clin Invest.

[2] Sanders TA, Vickers M, Haines AP (1981). Effect on blood lipids and haemostasis of a supplement of cod-liver oil, rich in eicosapentaenoic and docosahexaenoic acids, in healthy young men. Clin Sci (Lond).

[3] Burchard HU, Tischendorf FW (1988). Effect of cod liver oil administration on blood lipid levels, lipoprotein profile and bleeding time (Article in German). Z Ernahrungswiss.

[4] Simonsen T, Nordøy A, Sjunneskog C, Lyngmo V (1988). The effect of cod liver oil in two populations with low and high intake of dietary fish. Acta Med Scand.

[5] Weiner BH, Ockene IS, Levine PH (1986). Inhibition of atherosclerosis by cod-liver oil in a hyperlipidemic swine model. N Engl J Med.

[6] Popp-Snijders C, Schouten JA, de Jong AP, van der Veen EA (1984). Effect of dietary cod-liver oil on the lipid composition of human erythrocyte membranes. Scand J Clin Lab Invest.

[7] Brox J, Olaussen K, Osterud B, Elvevoll EO, Bjørnstad E, Brattebøg G, Iversen H (2001). A long-term seal- and cod-liver-oil supplementation in hypercholesterolemic subjects. Lipids.

[8] Ibrahim MA, Asuka E, Jialal I (2021). Hypercholesterolemia. https://www.ncbi.nlm.nih.gov/books/NBK459188/.

[9] Olsson AG, Pears J, McKellar J, Mizan J, Raza A (2001). Effect of rosuvastatin on low-density lipoprotein cholesterol in patients with hypercholesterolemia. Am J Cardiol.

[10] Jones P, Kafonek S, Laurora I, Hunninghake D (1998). Comparative dose efficacy study of atorvastatin versus simvastatin, pravastatin, lovastatin, and fluvastatin in patients with hypercholesterolemia (the CURVES study). Am J Cardiol.

[11] Jones PH, Davidson MH, Stein EA (2003). Comparison of the efficacy and safety of rosuvastatin versus atorvastatin, simvastatin, and pravastatin across doses (STELLAR* Trial). Am J Cardiol.

[12] (2021). Rosuvastatin oral: uses, side effects, interactions, pictures, warnings & dosing. https://www.webmd.com/drugs/2/drug-76701/rosuvastatin-oral/details.

[13] Nestel PJ (1990). Effects of N-3 fatty acids on lipid metabolism. Annu Rev Nutr.

[14] Nakatani T, Kim HJ, Kaburagi Y, Yasuda K, Ezaki O (2003). A low fish oil inhibits SREBP-1 proteolytic cascade, while a high-fish-oil feeding decreases SREBP-1 mRNA in mice liver: relationship to anti-obesity. J Lipid Res.

[15] Kim HJ, Takahashi M, Ezaki O (1999). Fish oil feeding decreases mature sterol regulatory element-binding protein 1 (SREBP-1) by down-regulation of SREBP-1c mRNA in mouse liver. A possible mechanism for down-regulation of lipogenic enzyme mRNAs. J Biol Chem.

[16] Xu J, Teran-Garcia M, Park JH, Nakamura MT, Clarke SD (2001). Polyunsaturated fatty acids suppress hepatic sterol regulatory element-binding protein-1 expression by accelerating transcript decay. J Biol Chem.

[17] Shen T, Xing G, Zhu J (2017). Effects of 12-week supplementation of marine omega-3 PUFA-based formulation omega3Q10 in older adults with prehypertension and/or elevated blood cholesterol. Lipids Health Dis.

